# Bacteria in *Ostreococcus tauri* cultures – friends, foes or hitchhikers?

**DOI:** 10.3389/fmicb.2014.00505

**Published:** 2014-11-07

**Authors:** Sophie S. Abby, Marie Touchon, Aurelien De Jode, Nigel Grimsley, Gwenael Piganeau

**Affiliations:** ^1^Institut Pasteur, Microbial Evolutionary GenomicsParis, France; ^2^CNRS, UMR 3525Paris, France; ^3^CNRS, UMR 7232, Biologie Intégrative des Organismes Marins, Observatoire OcéanologiqueBanyuls-sur-Mer, France; ^4^Sorbonne Universités, UPMC Université Paris 06, UMR 7232, BIOM, Observatoire Océanologique, Banyuls-sur-Mer, France

**Keywords:** phytoplankton, bacterial symbiosis, secretion system, illumina sequencing, bacterial diversity, microbiome, phycosphere, *Ostreococcus tauri*

## Abstract

Marine phytoplankton produce half of the oxygen we breathe and their astounding diversity is just starting to be unraveled. Many microbial phytoplankton are thought to be phototrophic, depending solely on inorganic sources of carbon and minerals for growth rather than preying on other planktonic cells. However, there is increasing evidence that symbiotic associations, to a large extent with bacteria, are required for vitamin or nutrient uptake for many eukaryotic microalgae. Here, we use *in silico* approaches to look for putative symbiotic interactions by analysing the gene content of microbial communities associated with 13 different *Ostreococcus tauri* (Chlorophyta, Mamilleophyceae) cultures sampled from the Mediterranean Sea. While we find evidence for bacteria in all cultures, there is no ubiquitous bacterial group, and the most prevalent group, *Flavobacteria*, is present in 10 out of 13 cultures. Among seven of the microbiomes, we detected genes predicted to encode type 3 secretion systems (T3SS, in 6/7 microbiomes) and/or putative type 6 secretion systems (T6SS, in 4/7 microbiomes). Phylogenetic analyses show that the corresponding genes are closely related to genes of systems identified in bacterial-plant interactions, suggesting that these T3SS might be involved in cell-to-cell interactions with *O. tauri*.

## INTRODUCTION

Eukaryotes acquired photosynthesis on multiple occasions from endosymbiosis (Archibald, 2012; De Clerck et al., 2012), resulting in an astounding phylogenetic diversity of phytoplanktonic eukaryotes (Not et al., 2012 for a review). The coexistence of so many species competing for the same resources does not fit theoretical prediction that in a stable environment, the best competitor wins, a puzzle coined as the “paradox of the plankton” (Hutchinson, 1961). However spatial and temporal environmental heterogeneities affect the environmental stability hypothesis (e.g., Levins and Culver, 1971), and interactions among competitors (e.g., Gross, 2008), as well as non-competitive interactions with other species (e.g., mutualism, commensalism) may increase their probability of coexistence. The “phycosphere” – the region immediately surrounding and influenced by phytoplankton cells – is an important bacterial habitat (Bell and Mitchell, 1972; Blackburn et al., 1998). Heterotrophic bacterial communities are sustained by phytoplankton exudates and play an important role in remineralization of nitrogen (N) and phosphate (P). Many protist algae are mixotrophic, gaining nutrients either by photosynthesis or by heterotrophy depending on environmental conditions (Flynn et al., 2013 for a review), adding further complexity to plankton community assemblages. Facilitative interactions have often been suggested by phycologists whose algal laboratory cultures were often most successful when they did not eliminate all bacteria from the cultures (Cole, 1982 for a review), and the generality of bacterial-seaweed associations is now well known (Goecke et al., 2010; Egan et al., 2013; Hollants et al., 2013 for reviews). For example, some protists are known to fix nitrogen or carbon via their cyanobacterial endosymbionts (reviewed in Nowack and Melkonian, 2010; Thompson and Zehr, 2013) and some chemical pathways mediating algal-bacterial interactions were identified (e.g., Seyedsayamdost et al., 2011; Patzelt et al., 2013; Syrpas et al., 2014). Whether some phytoplanktonic eukaryotes evolved specific interactions with bacteria beyond ecological facilitation is still a matter of debate, and little information is available on close algal-bacterial interactions for unicellular eukaryotes. The study of interactions between planktonic microbes has been long hampered by our lack of knowledge of these unicellular organisms, especially for the smallest sized planktonic eukaryotes, the picoeukaryotes (cell diameter size <2 μm). These microorganisms often lack morphologically informative characters and are difficult to isolate and maintain in culture. For example, species of the genus *Ostreococcus* are hard to discriminate, and it is thus difficult to study species-specific interactions between partners one cannot identify (Subirana et al., 2013).

Early observations of physical attachment between some diatom species and bacteria date back to the first cytological observations (e.g., the diatom *Skeletonema costatum* in Droop and Elson, 1966). A pioneering study of bacteria-phytoplankton interactions screened the bacterial content of microalgal cultures by standard microbiological techniques (Berland and Maestrini, 1969). With the development of molecular biology tools, ribosomal RNA genes sequencing and barcoding approaches allowed establishing links between phytoplankton and bacterial community dynamics in natural communities (Fukami et al., 1992; Rooney-Varga et al., 2005) and culture collections of diatoms and dinoflagellates (Schäfer et al., 2002; Jasti et al., 2005; Sapp et al., 2007). Recently, integrative approaches associating sequence-based identification of cells, cytometry, cell sorting and tracer experiments with ^15^N and ^13^C, demonstrated a mutualistic interaction between N_2_-fixing cyanobacteria and a phytoplanktonic prymnesiophyte (Thompson et al., 2012). Cell sorting and single cell sequencing of natural isolates now enables to identify candidate bacteria–protist partnerships without cultivation, and these techniques promise to uncover overlooked interactions (Martinez-Garcia et al., 2012).

**FIGURE 1:**
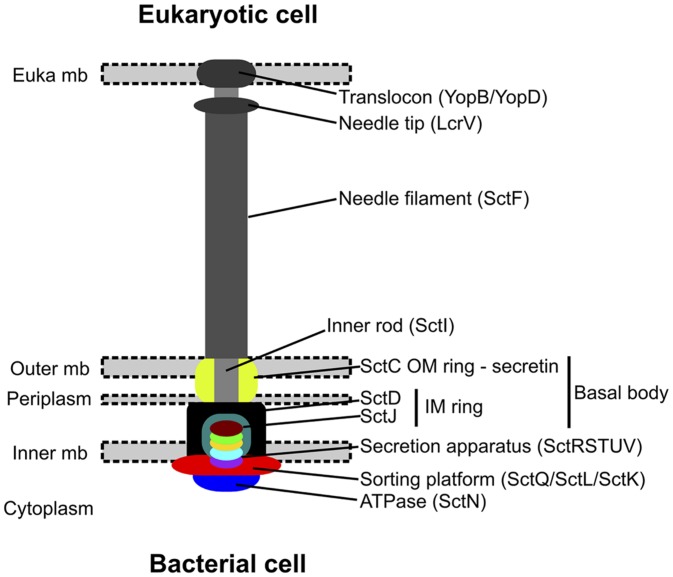
**Assembled structure of the type 3 secretion system (modified from Abby and Rocha, 2012).** This system is made of an extracellular part for close contact with host cell, and a membrane-associated part containing the core secretion apparatus. The components with bright colors correspond to the most conserved components, and are thus the one searched by sequence similarity to assess the system’s presence in the assembled sequences.

The development of next generation sequencing now enables to sequence microalgae and associated microbiomes and to investigate the molecular toolkit of putative interactions from gene content analyses. For example, symbiosis involving nitrogen fixation requires the presence of the nitrogenase operon in the bacterial partner, and complementation for vitamin biosynthesis requires the presence of the genes responsible for the vitamin B pathway in the bacteria along with suitable algal uptake mechanisms (**Table 1**). In some plant-bacterium interactions, physical cell-to cell-interactions occur via specialized protein secretion systems such as the type 3 secretion systems (T3SS; Kosarewicz et al., 2012), or the T4SS (Smillie et al., 2010). These systems are sophisticated molecular needles that enable the translocation of bacterial effectors from the bacterial cytoplasm to the eukaryotic cell (**Figure 1**). These systems are involved in both antagonistic and beneficial interactions between bacteria and eukaryotes.

**Table 1:** The Molecular toolkit of bacteria – eukaryote interactions: examples of bacterial target genes for screening genomes and metagenomes.

Type	Name of Pathway	Phylogenetic spread	Number of genes	Reference
Directed secretion of bacterial proteic effectors	Type 3 secretion system Type 4 secretion system Type 6 secretion system	Proteobacteria and Chlamydiae Proteobacteria Mostly proteobacteria	20–25 12 15	Galan and Wolf-Watz (2006) Backert and Selbach (2008), Bardill et al. (2005) Pukatzki et al. (2006)

Metabolic complementation on Nitrogen	Nitrogenase – diazotrophy	Proteobacteria, Cyanobacteria, Firmicutes, Euryarchaea	Operon of five genes: NifHDKEN	Raymond et al. (2004), Seefeldt et al. (2009)

Metabolic complementation on vitamin synthesis	B12 B1, B2	Euryarchea Proteobacteria Firmicutes Actinomycete Cyanobacteria	3–11 genes	Helliwell et al. (2011), Wagner-Döbler et al. (2009), Croft et al. (2005)

While picoalgae of the genus *Ostreococcus* are distributed worldwide (Piganeau and Moreau, 2007; Demir-Hilton et al., 2011), *Ostreococcus tauri*, first isolated from a French Mediterranean coastal lagoon and described as the smallest eukaryotic species known (Courties et al., 1994; Chrétiennot-Dinet et al., 1995) has been found mainly in coastal regions and lagoons (Subirana et al., 2013). Within a 3-year period (2006–2008) of sustained effort, we could isolate 17 new wild-type clonal lines of *O. tauri* and characterize them with a few genetic markers (Grimsley et al., 2010). Interestingly, none of the *O. tauri* cultures we retained were completely axenic despite initial treatment with antibiotics. Here, we postulate that the bacterial microbiome consistently present in successful *O. tauri* cultures could be required for the health of these cultures. We present an analysis of the bacterial microbiome associated with these cultures and apply recently developed tools to mine the *O. tauri* microbiome for protein secretion systems involved in bacteria-eukaryotes interaction.

## MATERIAL AND METHODS

### BIOLOGICAL DATA

We analyzed data from 13 *O. tauri* strains that were sampled from surface water in five locations by the North-West Mediterranean sea previously described (Blanc-Mathieu et al., 2013). Cultures were isolated by serial filtrations, addition of Keller’s salts as a supplement (Keller et al., 1987), and growth in a culture chamber in the laboratory. These strains were established from clonal culture by plating out in gelled medium and re-inoculating cells picked from a single colony in liquid medium to obtain cell densities above 10^7^ ml^-1^. One strain was the control *O. tauri* laboratory strain RCC4221, cloned from the RCC745 culture (Courties et al., 1994), and the other 12 were isolated more recently as described in Grimsley et al. (2010). Despite the treatment by antibiotics as described in Grimsley et al. (2010); kanamycin 20 μg/ml, penicillin 25 μg/ml, and neomycin 20 μg/ml final concentrations, none of the strains were found to be completely axenic. This is the case not only for the 13 strains analyzed here, but also for 100s of other isolations made by plating out for single algal cells, including *O. mediterraneus, Micromonas sp.* and *Bathycoccus prasinos* (Nigel Grimsley, unpublished observations). Three micrograms of total DNA was extracted from each culture as previously described (Derelle et al., 2006). Genomic DNA of the strains was randomly sheared into ∼250-bp fragments. The libraries created from these fragments were sequenced on an Illumina GAIIx and Hiseq system at the Joint Genome Institute^1^ Community Sequencing Program (CSP-129). Sequence data for strains RCC1108, RCC1114, RCC1115, RCC1116, RCC1558, RCC1559, and RCC4221 were 76 bp paired-end reads (depth of coverage for the algal genome 160-fold to 340-fold) and sequence data of strains RCC1110, RCC1112, RCC1117, RCC1118, RCC1123, and RCC1561 were 101 bp paired-end reads (depth of coverage for the algal genome 780-fold to 1130-fold).

### EXTRACTION OF BACTERIAL SEQUENCES AND TAXONOMIC AFFILIATION

Paired-end data of each strain were mapped to the reference nuclear genome of *O. tauri* (GenBank accession numbers: CAID01000001-CAID01000020, (Blanc-Mathieu et al., under review) with the Burrow-Wheeler Aligner (BWA) with parameters *n*= 6 *l*= 35 *k*= 3 *e*= 3 (Li and Durbin, 2009). Read pairs with no read mappings to the genome sequence were identified based on SAM flags and extracted in fastq files with the seqtk package^2^. Paired-end reads were assembled with Velvet (Zerbino and Birney, 2008) using default parameters and various *k-mer* sizes. The assembly with the highest median contig length (N50) was retained.

To remove remaining *Ostreococcus* sequences that might have been too divergent to be mapped on to the reference sequence, contigs with more than 80% amino-acids identity over 90 bp with available Mamiellales coding sequences were discarded using the PRASINOID interface^3^ (Vaulot et al., 2012). The remaining contigs were analyzed via MG-RAST (Meyer et al., 2008) on the Refseq protein database. Taxonomic affiliations for each microbiome were downloaded from the MG-RAST server and analyzed using in-house scripts to retrieve all contigs with an alignment against Refseq longer than 30 amino-acids and with more than 80% sequence identity.

The assembled bacterial contigs for each culture can be downloaded from http://www.obs-banyuls.fr/piganeau/publications/data/.

### ESTIMATION OF UBIQUITY AND ABUNDANCE OF BACTERIA

The abundance of each bacterial group was measured as the sum of reads affiliated to one group, divided by the total number of affiliated reads for each microbiome. Ubiquity was defined as the number of occurrences of a genus in the 13 metagenomes.

To estimate the bacteria to *Ostreococcus*-cells ratio, we assumed that the number of reads affiliated to *Ostreococcus*, *r_O_* is equal to the product of the number of *Ostreococcus* cells in the sample, *C_O_*, by the genome size, *G_O_*, by a constant α*_O_* (representing the product of the DNA extraction efficiency and the sequencing efficiency):

(1) $$R_{O}=\alpha_{O}G_{O}C_{O}\,$$

The number of reads affiliated to bacteria, *R_B_*, is equal to the sum of the product of *C_Bi_* the number of bacterial cells from each bacterial strain *i*, by *G_Bi_* their average genome size, by a constant α*_Bi_*. Assuming an equal α and genome size between bacteria (marine bacteria have a genome in the 2–7 Mb size range), the complete number of bacterial reads can be expressed as the product of *C_B_*, *G_B_*_,_ and α*_B_*.

$$R_{B}=\sum_{i}\alpha_{Bi}G_{Bi}C_{Bi}\approx \alpha_{B}G_{B}C_{B}$$

From these two equations we can estimate the ratio of bacterial to *Ostreococcus* cells as a function of two parameters: the relative genome size differences, the relative number of reads and the relative α parameter between bacteria and *Ostreococcus* DNA.

(2) $$\frac{C_{B}}{C_{O}}=\frac{\alpha_{O}G_{O}R_{B}}{\alpha_{B}G_{B}R_{O}}$$

Statistical analyses and ubiquity abundance plots were done with R^4^.

### SEARCH FOR NITROGENASES

To screen for the presence of nitrogenases, we used the amino-acid sequences of the different types of nitrogenases described in Raymond et al., (2004). We processed the output of the blastx of this dataset against the assemblies to retain all hits with amino-acid identity greater or equal to 60% and total alignment length higher than 100 amino-acids. Ten cultures contained hits with these criteria. Further analysis of theses hits did not confirm that the encoding genes were nitrogenases, but genes belonging to related gene families, like hydrogenases, so that we did not proceed to further analysis.

### IDENTIFICATION AND PHYLOGENETIC ANALYSIS OF PROTEIN SECRETION SYSTEMS

Tools were recently developed to identify type 3, type 4, and type 6 secretion systems from similarity search of essential components and gene content/gene organization criteria (Abby and Rocha, 2012; Gama et al., 2012; Guglielmini et al., 2014). They were used with the MacSyFinder framework (Abby et al., 2014) to detect these systems in the assembled contigs of the microbiomes. Phylogenetically relevant components of T3SS detected in the microbiome were analyzed (i.e., SctJ, SctN, SctQ, SctR, SctS, SctT, SctU, and SctV; see **Figure 1**). Their sequences were introduced into the appropriate pre-existing gene families (dataset of Abby and Rocha, 2012). We aligned their sequences with Muscle (default parameters) and selected informative sites with BMGE (BLOSUM30 similarity matrix, gap rate cut-off = 0.20, sliding window size = 3, entropy score cut-off = 0.5; Edgar, 2004; Criscuolo and Gribaldo, 2010). Then, these alignments were concatenated, and a phylogenetic tree including microbiome sequences was built with RAxML (Le and Gascuel matrix + 4-categories-discretized Gamma distribution for rate variation among sites + empirical frequencies of amino-acids) with 100 rapid bootstraps (Stamatakis, 2006; Le and Gascuel, 2008).

## RESULTS

### WHICH BACTERIA LIVE IN *O. tauri* CULTURES?

**FIGURE 2:**
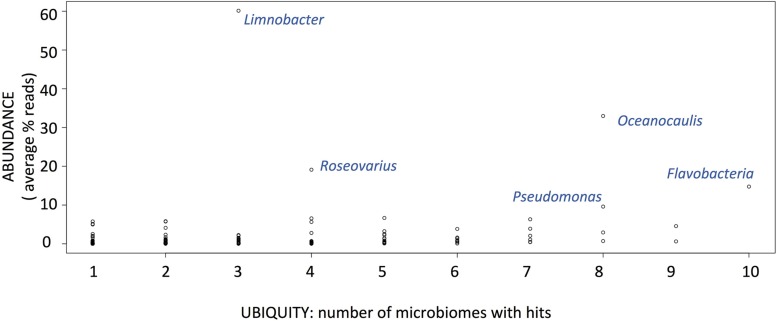
**Ubiquity versus abundance (average % of reads across microbiomes where the group is present) of bacterial groups after MGRAST analysis of the 13 microbiomes**.

MG-RAST taxonomic affiliation of contigs based on sequence identity to the Refseq protein database listed 149 distinct bacterial taxonomic affiliations at the genus level. A few contigs were assigned to higher taxonomic ranks like alphaproteobacteria or gammaproteobacteria. We estimated the average abundance of each group as the proportion of reads affiliated to a bacterial group to the total number of reads assigned to bacteria. Based on these estimates, we found no bacterial group to be both ubiquitous and abundant throughout the 13 microbiomes (**Figure 2**). The most ubiquitous bacterial group is *Flavobacterium* and is found in 10 out of 13 cultures, with an average abundance of 18% of reads in the 10 microbiomes. The most abundant group of bacteria is *Limnobacter* as this genus is represented by 60% of the reads in the three microbiomes where it was detected. Other abundant groups are *Pseudomonas*, *Roseovarius,* and *Oceanocaulis* (**Figure 2**), each group represent more than 10% of the reads. The most abundant bacterial groups for each microbiome are reported in **Table 2**. They belong to seven different genera for the 13 microbiomes; *Flavobacterium*, *Pseudomonas,* and *Limnobacter* dominate in the six microbiomes with more than one bacterium for 10 *O. tauri* cells, while *Sphingomonas*, *Robiginitalea*, *Oceanocaulis,* and *Roseovarius* are the most abundant bacterial groups in the seven microbiomes with less than one bacterium for 10 *O. tauri* cells (see below). These genera belong to the Bacteroidetes and Proteobacteria phyla, with three orders present for the latter: alphaproteobacteria, betaproteobacteria, and gammaproteobacteria.

**Table 2:** Number of reads assigned to *Ostreococcus tauri* and bacteria for each cultured strain and estimation of the number of bacterial cells for 10 *O. tauri* cells from Eqn (2) for the most abundant bacteria.

Strain	*Ostreococcus tauri* reads (x10^5^)	Total Bacterial reads (x10^5^)	Taxonomic Affiliation^+^ of most abundant bacteria [% reads]	*C_B_*/(10 *C_O_*)
RCC1108*	1161	185	*Pseudomonas* (γP) [70]	3
RCC1110*	484	69	*Flavobacterium* (F) [59]	2
RCC1112*	840	299	*Limnobacter* (βP) [43]	4
RCC1114*	331	34	*Limnobacter* (βP) [73]	2
RCC1115	459	3	*Sphingomonas* (αP) [13]	0.02
RCC1116	448	22	*Robiginitalea* (F) [58]	1
RCC1117*	1037	248	*Limnobacter* (βP) [66]	4
RCC1118	1219	6	*Oceanicaulis* (αP) [80]	0.1
RCC1123	1151	72	*Oceanicaulis* (αP) [41]	1
RCC1558	423	2	*Oceanicaulis* (αP) [47]	0.05
RCC1559	235	3	*Oceanicaulis* (αP) [98]	0.3
RCC1561*	1096	153	*Flavobacterium* (F) [38]	1
RCC4221	301	14	*Roseovarius* (αP) [57]	1

Genome size of *O. tauri* is 13 Mb and bacterial genome size was assumed to be 4 Mb.

*T3SS detected from microbial assemblage.

*^+^α, β, γ P, alpha, beta, gamma Proteobacteria; F, Flavobacteria.*

### HOW MANY BACTERIA PER *O. tauri* CELL?

We used the number of reads affiliated to *O. tauri* and to the most abundant bacterial groups to estimate the number of bacterial to microalgal cells using Eqn (2). This estimation relies on the assumption that the DNA extraction protocol and the sequencing are not biased towards *O. tauri* or bacteria. In a 45 marine bacterial genomes dataset, the average genome size was estimated to be ∼4 Mb (Moran and Armbrust, 2007). We used this value as a proxy for genome size of bacteria associated to *O. tauri* strains. Since marine bacterial genome size varies between 2 and 7 Mb, we do not expect more than a twofold difference in our estimate. The number of bacteria for 10 *O. tauri* cells varies by two orders of magnitude between cultures: from 0.02 to 4 (**Table 2**).

### LOOKING FOR BACTERIAL FACTORS INVOLVED IN INTERACTIONS WITH EUKARYOTES

Type 3 secretion systems have evolved and diversified into recognizable sub-types to interact with different kinds of eukaryotic cells (animal vs. plant), and participate in different types of interaction with eukaryotes (antagonistic vs. beneficial; Troisfontaines and Cornelis, 2005; Abby and Rocha, 2012). The human pathogen *Salmonella* uses two types of T3SS (SPI-1 and SPI-2) at different stages of host infection (Valdez et al., 2009), while nitrogen-fixing Rhizobiales use a particular T3SS (Rhizobiales type) to establish symbiotic interactions with host plants (Dai et al., 2008; Kambara et al., 2009). Therefore, the presence of T3SS components in a bacterial dataset is a hallmark of bacterium-eukaryote interactions, and the phylogenetic typing of these components can help identifying the kind of interaction, and the type of targeted eukaryotic cell. Among T4SS – classically dedicated to conjugation – some are involved in plant pathogenesis (e.g., tumor formation, Pitzschke and Hirt, 2010) but also in plant symbiosis (Hubber et al., 2004). T6SS allow the translocation of effectors from bacteria to eukaryotic cells in antagonistic relationships, but were also proved to target bacteria for bacterial competition (Pukatzki et al., 2007; Hood et al., 2010; Kapitein and Mogk, 2013). T6SS are virulence factors for several phytopathogens, and were observed in plant symbionts genomes (Amadou et al., 2008; Wu et al., 2008). We looked for signs of these putative factors of bacterium-eukaryote interaction in the microbiota associated to *O. tauri*. Using both gene content and close linkage distance between genes as criteria for the detection of T4SS and T6SS (Gama et al., 2012; Guglielmini et al., 2014), there was no evidence of T4SS seemingly involved in protein secretion, but we could detect six occurrences of putative T6SS in four different microbiomes (Table S1).

**FIGURE 3:**
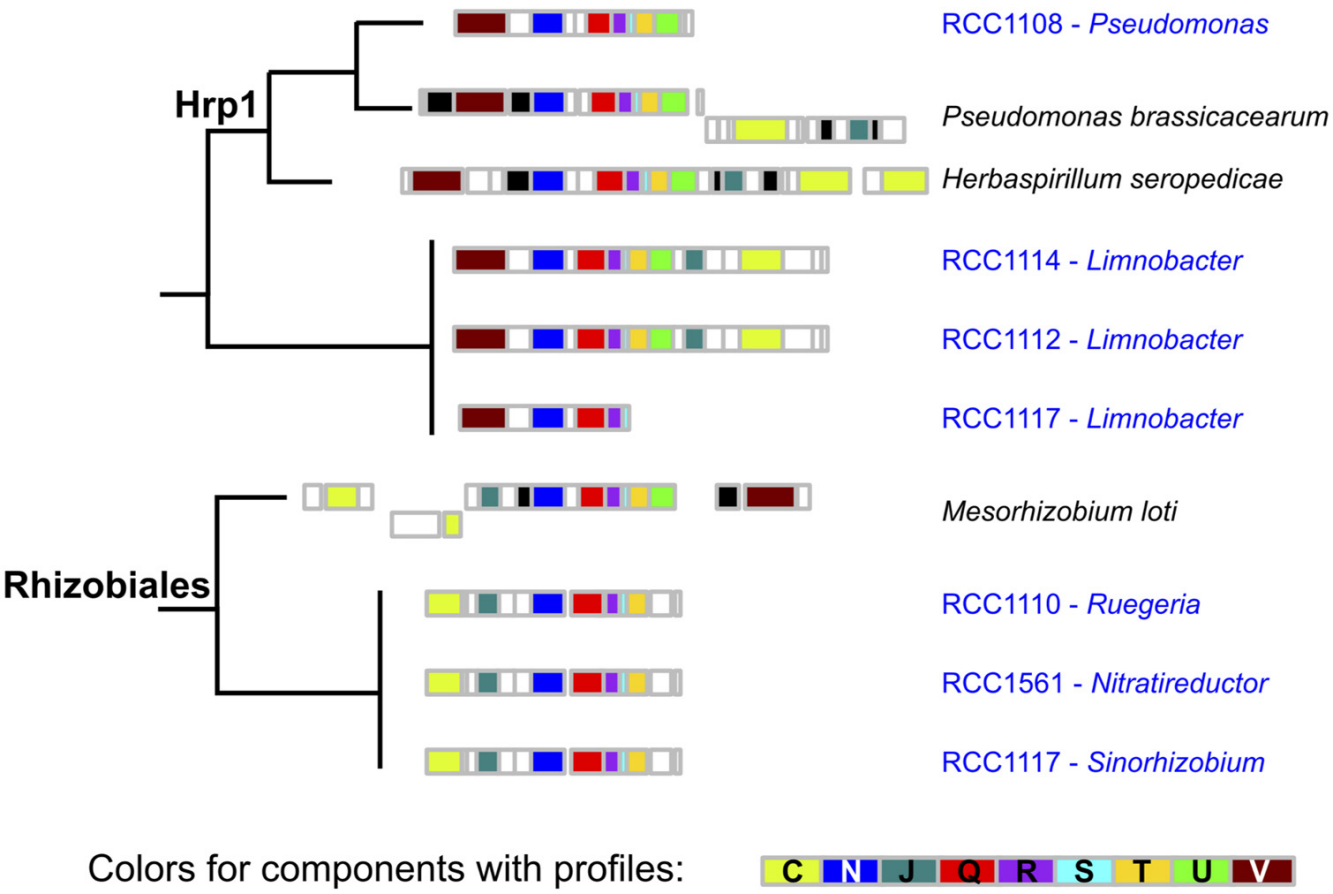
**Genetic architecture of microbiome T3SS.** The genetic architecture of T3SS detected in microbiomes’ contigs and of the closest systems found in a complete genome is displayed along the corresponding phylogeny. The color of boxes corresponds to the colors in **Figure 1**.

**FIGURE 4:**
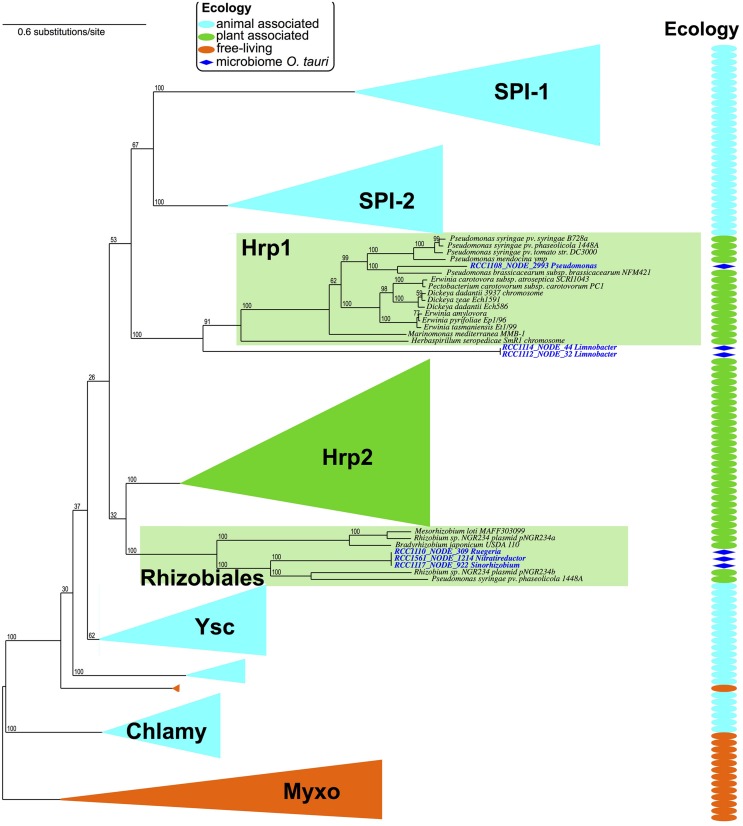
**The phylogeny of T3SS reflects host-cell type.** The T3SS detected in *O. tauri* microbiomes were integrated in a reference phylogeny of the T3SS (Abby and Rocha, 2012). T3SS phylogeny shows a diversification of T3SS into host-cell adapted sub-types: animal associated, and plant associated (“ecology” panel, and column). While most of these sub-types are mostly involved in pathogenic interactions with plants or animals, some Hrp1 T3SS and all Rhizobiales T3SS are involved in symbiotic associations with plants.

Using the same kind of detection methods for type 3 secretion systems (Abby and Rocha, 2012), we could detect seven occurrences of putative T3SS in contigs from six different microbiomes (**Figure 3**). We took advantage of a previous study to sub-type T3SS: we included the T3SS’ components detected in *O. tauri*’s microbiome in a reference phylogeny annotated with T3SS sub-types and corresponding host types (Figure 4 of Abby and Rocha, 2012). Interestingly, detected T3SS consistently placed within, or as sister-groups of plant-associated T3SS sub-types with high rapid bootstrap supports (**Figure 4**). A first sub-type placed within the “Hrp1” T3SS family, which includes many systems of plant pathogens and some involved in plant symbiosis (i.e., *Pseudomonas syringae, Dickeya dadantii*). This system, whose contig was assigned to the *Pseudomonas* genus, was found in a single microbiome and showed sequence and genetic architecture highly similar to the T3SS observed in the genome of *P. brassicacearum*, a root-associated plant symbiont (Ortet et al., 2011). Three T3SS occurrences seemed to correspond to a single system that placed within the clade of plant-symbionts, Rhizobiales T3SS. This system showed high similarity with a *Mesorhizobium* system (Rhizobiales species, 50–70% identity in a Blast analysis). The three corresponding contigs were attributed to three different alphaproteobacterial species: 2 Rhizobiales and 1 Rhodobacterales. Intriguingly, a third type of T3SS found in two microbiomes fell outside of the defined T3SS families, but constituted a sister-group of the Hrp1 family (**Figure 4**). The closest system found in the phylogeny was that of *Herbaspirillum seropedicae*, a plant symbiont, and both taxonomic attribution of contigs and similarity searches of the system using Blast and MG-RAST pointed at the species *Limnobacter sp.*, a Burkholderiales.

Overall, one of the detected T6SS gene cluster was found on a contig assigned to the *Pseudomonas* genus, in the same microbiome where a *Pseudomonas* T3SS was inferred (RCC1108, see Table S1; **Figure 4**). Five of the six detected T6SS were found in three microbiomes showing also evidence of T3SS gene clusters.

## DISCUSSION

Despite the efforts of many laboratories over the last century to define the media and growth conditions required for different marine algae, most have so far remained recalcitrant to growth in a completely defined medium, and require seawater to grow over essential extra nutrients (e.g., phosphate and nitrate). Seawater is a complex solution of chemicals and organisms that can vary considerably in its composition between geographically distant regions, complicating the development of appropriate culture media. Collections are thus often located in marine biology laboratories close to coastlines. The majority of algal cultures cannot be maintained axenically, rendering physiological analyses of their nutritional requirements more difficult. Indeed, many unicellular algae are mixotrophic, and can satisfy part of their nutritional requirements by ingesting bacteria.

Despite antibiotics treatments and the isolation of single cells from colonies in soft agarose, none of the *O. tauri* strains were axenic. This was the case not only for the 13 strains analyzed here, but also for 100s of other isolations made by plating out for single algal cells (unpublished observations). In addition, bacterial cultures issued from media of such cultures as well as observations of algal strains by flow cytometry almost always confirmed the presence of common seawater bacteria (unpublished data). These observations strongly suggest that either *O. tauri* adhere to bacterial cells during the cultivation process, or that *O. tauri* require some unidentified substance from bacterial cells for growth. Recent experimental evidence and genome analysis suggests that *O. tauri* is vitamin B12-dependent (Helliwell et al., 2011). We provided three vitamins (thiamine [B1]), biotin [H], and cyanocobalamin [B12]) as described for standard Keller’s medium (Keller et al., 1987), making unlikely the selection of bacteria for vitamin B12 production. In order to identify putative supplements required by isolated *O. tauri* cells for growth, future work could focus in the use of sterile artificial seawater, and step-by-step introduction of candidate substances. But considering the great deal of effort already put in attempts to define suitable culture media for this kind of algae (Keller et al., 1987), it might be more efficient to first isolate associated bacteria and investigate their influence on the physiology of the microalgae (Le Chevanton et al., 2013).

The bioinformatic analyses performed here confirmed the presence of a diverse collection of common marine bacteria in *O. tauri* cultures. *Flavobacteria* was largely found in our samples. This important class of *Bacteroidetes* often constitutes a significant portion of marine microbial communities and has been reported in microalgal cultures (Berland and Maestrini, 1969; Mann et al., 2013). Similarly, *Bacteroidetes* have also been reported in the surface waters of NW Mediterranean sea (Lami et al., 2009). *Flavobacteria* are found both free-living and attached to organic aggregates and are considered as major mineralizers of organic matter (Kolton et al., 2013). Interestingly, the type 3 and type 6 secretion systems have been detected in the microbiomes with higher bacterial prevalence. Microbiomes for strains that have a higher ratio of number of bacteria to *O. tauri* cells (**Table 2**), are more likely to contain a detectable T3SS (Wilcoxon signed rank test *p* < 0.01). The taxonomic affiliation of the contigs containing a predicted T3SS correspond to the most abundant bacterial group in the microbiome except for RCC1110 and RCC1561, whose T3SS contigs were assigned to alphaproteobacteria while the most abundant bacterial group is a *Flavobacterium*. The contigs containing the secretion systems were attributed to genera with evidence of species interacting with eukaryotes via protein secretion systems. Finally, no single strain of bacterium was found in all of the *O. tauri* cultures. Since all these bacteria were isolated from the same host species, it could seem unlikely at first that very specific physical interactions or nutritional requirements exist between *O. tauri* and its microbiome. However, several biases could partially explain the heterogeneity observed between the microbiomes both in terms of taxonomic diversity, and therefore gene content. Firstly as the effective detection of the secretions systems rely on sequence similarity search and the genetic organization of their components, it heavily depends on the sequencing and assembly quality. In the context of NGS approaches for metagenomics, whose short reads are difficult to assemble, it is likely that we missed occurrences of systems due to contig assembly errors and biases. Secondly, the filtration steps in *O. tauri* isolation is likely to sort out aggregates of bacterial cells, and as a consequence, some bacteria of interest to understand the growth of *O. tauri*. Finally, antibiotics treatment had also an impact on the bacterial populations we analysed in this study, and this might also explain some of the heterogeneity in terms of bacterial diversity and gene content. To specifically find preferred associated bacteria we should repeat this work without using antibiotics, even if these conditions, it may be difficult to isolate *Ostreococcus* strains.

In conclusion, we provide evidence of pervasive bacterial presence in *O. tauri* cultures, despite initial antibiotic treatment. We provide evidence for putative plant-associated T3SS in six microbiomes, and several cases of T6SS in four microbiomes (three displayed both systems). For now there are no studies showing a clear association between T6SS sub-types and their function, thus it is hard to define from genomic analyses whether the systems we detected are targeting bacteria or eukaryotes. But in both cases, they might be parts of interactions between bacteria and eukaryotes, even indirectly: *via* bacterial competition, T6SS were found to serve as colonization factors in the plant pathogen *Agrobacterium tumefaciens*, (Ma et al., 2014) and to provide plants a protection against pathogens in *P. fluorescens* (Decoin et al., 2014). On the other hand, the analysis of detected T3SS gave a clearer picture as it clearly shows they are involved in interaction with plant cells. The three sub-types they belong to – or the groups they are closer to, all contain systems typical of plant symbiosis, and pathogenicity in the case of the Hrp1 group. Further experimental work is required to determine the impact of these secretion systems in *O. tauri* growth, while keeping in mind that interactions are dynamic, and that the same bacteria may change between “friend,” “foe,” or “hitch-hiker” over time or environmental conditions (Andrade-Domínguez et al., 2014).

## Conflict of Interest Statement

The Review Editor, Fabrice Not, declares that, despite having collaborated with author, Nigel Grimsley, the review process was handled objectively and no conflict of interest exists. The authors declare that the research was conducted in the absence of any commercial or financial relationships that could be construed as a potential conflict of interest.

## Footnotes

1 http://www.jgi.doe.gov/

2 https://github.com/lh3/seqtk

3 http://www.obs-banyuls.fr/prasinoidtest

4
