# Supplementary material for: Bacteria in Ostreococcus tauri cultures – friends, foes or hitchhikers?
Source: Front Microbiol. 2014 Nov 7;5:505. doi: 10.3389/fmicb.2014.00505 (PMC4224133; doi:10.3389/fmicb.2014.00505)
Supplement: Supplementary file 1 [file Data_Sheet_1.DOCX]

**Supplementary Table S1:** *O. tauri* cultures in which T3SS and/or T6SS have been detected.

| **Microbiome** | **System** | **Core gene content** | **Sub-type (for T3SS)** | **Attributed taxon** |
| --- | --- | --- | --- | --- |
| RCC1108 | T3SS | 7/9 | Hrp1 | *Pseudomonas* |
|  | T6SS | 10/14 |  | *Burkholderia* |
|  | T6SS | 11/14 |  | *Pseudomonas* |
|  | T6SS | 9/14 |  | *Pseudomonas* |
| RCC1110 | T3SS | 6/9 | Rhizo | *Ruegeria* |
| RCC1112 | T3SS | 9/9 | out-Hrp1 | *Limnobacter* |
|  | T6SS | 8/14 |  | - |
| RCC1114 | T3SS | 9/9 | out-Hrp1 | *Limnobacter* |
|  | T6SS | 8/14 |  | Azoarcus |
| RCC1117 | T3SS | 5/9 | out-Hrp1 | *Limnobacter* |
|  | T3SS | 6/9 | Rhizo | *Sinorhizobium* |
| RCC1561 | T3SS | 6/9 | Rhizo | *Nitratireductor* |
| RCC2009 | T6SS | 7/14 |  | - |
